# Supplementary material for: Predicting diabetes mellitus metabolic goals and chronic complications transitions—analysis based on natural language processing and machine learning models
Source: PLoS One. 2025 Apr 15;20(4):e0321258. doi: 10.1371/journal.pone.0321258 (PMC11999128; doi:10.1371/journal.pone.0321258)
Supplement: S1 File — (DOCX) [file pone.0321258.s001.docx]

***Appendix 1. List of ICD-10 codes use to characterize complications.***

| Complication | ICD-10 | Description |
| --- | --- | --- |
| Retinopathy | H30 | Chorioretinal inflammation |
|  | H31 | Other disorders of choroid |
|  | H32 | Chorioretinal disorders in diseases classified elsewhere |
|  | H33 | Retinal detachments and breaks |
|  | H34 | Retinal vascular occlusions |
|  | H35 | Other retinal disorders |
|  | H36 | Retinal disorders in diseases classified elsewhere |
| Cerebrovascular disease | G450 | Vertebro-basilar artery syndrome |
|  | G451 | Carotid artery syndrome (hemispheric) |
|  | G452 | Multiple and bilateral precerebral artery syndromes |
|  | G453 | Amaurosis fugax |
|  | G454 | Transient global amnesia |
|  | G458 | Other transient cerebral ischaemic attacks and related syndromes |
|  | G459 | Transient cerebral ischaemic attack, unspecified |
|  | G46 | Vascular syndromes of brain in cerebrovascular diseases |
|  | I60 | Subarachnoid haemorrhage |
|  | I61 | Intracerebral haemorrhage |
|  | I62 | Other nontraumatic intracranial haemorrhage |
|  | I63 | Cerebral infarction |
|  | I64 | Stroke, not specified as haemorrhage or infarction |
|  | I65 | Occlusion and stenosis of precerebral arteries, not resulting in cerebral infarction |
|  | I66 | Occlusion and stenosis of cerebral arteries, not resulting in cerebral infarction |
|  | I67 | Other cerebrovascular diseases |
|  | I68 | Cerebrovascular disorders in diseases classified elsewhere |
|  | I69 | Sequelae of cerebrovascular disease |
| Chronic Kidney Disease | N03 | Chronic nephritic syndrome |
|  | N05 | Unspecified nephritic syndrome |
|  | N07 | Hereditary nephropathy, not elsewhere classified |
|  | N19 | Unspecified kidney failure |
|  | N25 | Disorders resulting from impaired renal tubular function |
|  | N18 | Chronic kidney disease (CKD) |
|  | N181 | CKD, stage 1 (GFR of at least 90mL/min) |
|  | N182 | CKD, stage 2 (GFR of at least 60-89mL/min) |
|  | N183 | CKD, stage 3 (GFR of at least 30-59mL/min) |
|  | N184 | CKD, Stage 4 (GFR of at least 15-29mL/min) |
|  | N185 | CKD, Stage 5 (GFR of 15mL/min or lower) |
