# Supplementary material for: Predicting diabetes mellitus metabolic goals and chronic complications transitions—analysis based on natural language processing and machine learning models
Source: PLoS One. 2025 Apr 15;20(4):e0321258. doi: 10.1371/journal.pone.0321258 (PMC11999128; doi:10.1371/journal.pone.0321258)
Supplement: S2 File — (DOCX) [file pone.0321258.s002.docx]

***Appendix 2.*** Details on the NLP algorithm

A2.1. ***Bag-of-Words (BoW) method.***

In this approach, the methodology includes several preprocessing steps to ensure that the text is in a standardized format for further analysis. The preprocessing steps are as follows: (i) All text is converted to lowercase to avoid any discrepancies due to case sensitivity. (ii) Any URLs present in the text are removed to eliminate irrelevant information. (iii) Common abbreviations are replaced and standardized to their full forms to enhance text clarity. (iv) All punctuation marks in the text are removed to focus solely on relevant words. (v) All digits present in the text are removed as they do not contribute to the analysis. (vi) Words with less than three characters are removed from the text as they are considered less informative. (vii) A custom dictionary, including Spanish words, medical terms, and a personalized manual dictionary, is used to perform spelling corrections on the text. (viii) The text is tokenized into individual words for further processing. (ix) Common stop words that do not add significant meaning to the analysis are removed. (x) The lemmatization process is applied to convert each word to its base or root form, reducing redundancy, the dimensionality of the data, and increasing efficiency. Finally, (xi) a stemming process is performed on each word to further simplify and standardize the vocabulary. By applying these preprocessing steps, the approach aims to ensure that the text data is in an appropriate and consistent format for subsequent analysis using the BoW methodology.

After preprocessing, the BoW methodology is applied for the characterization phase. BoW transforms each text into a set of numerical vectors by counting the frequency of relevant words identified based on the specific study case. These relevant word sets are defined in advance for each study.

In the classification phase, a score and a reliability index are calculated based on the frequency and occurrence of the relevant words in the text. A higher reliability index (greater than 1) indicates a lower risk of mislabeling, whereas a reliability index in the range [0,1] suggests a higher risk of assigning an incorrect label to the text.

To account for potential labelling uncertainty, all scores greater than zero are ranked in descending order to obtain a cumulative sum. The optimal point of the score is then determined using the elbow criterion.

The following are the equations for calculating the score and its respective reliability:

$$Score = sum(BoW\_CountVector)*len\left( words \right)^{2}$$

$$Reliability Index =\frac{Abs\left( Reg_{Score}- Border_{score} \right)}{Border\_Tolerance}$$

Finally, clinical experts from the research team validated a sample of around 100 records to assess the quality of the match. The team reported findings that helped to improve the dictionary and repeat the exercise. This procedure was carried on twice.


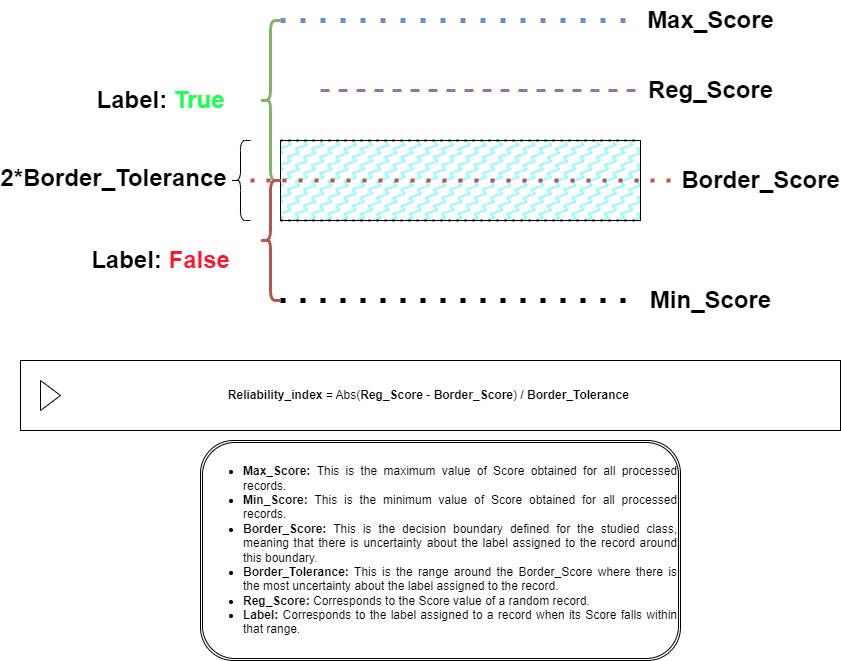


By employing these pre-processing steps and the BoW methodology, the approach aims to ensure the accurate and reliable classification of the texts based on the defined sets of relevant words for each case study.

A2.2. ***Software***

We used the following libraries in R to implement the NLP pipeline:

- TensorFlow (import tensorflow as tf): Load and run neural network models stored in .h5 format for deep learning-based model predictions (Keras).

- Dash (import dash, from dash import html, dcc): Create the interactive graphical user interface (GUI) for the web application.

- Plotly (import plotly.graph_objects as go, import plotly.express as px): Generate interactive plots such as Sankey diagrams, pie charts and bar charts to visualise prediction results.

- Dash Bootstrap Components (import dash_bootstrap_components as dbc): Use Bootstrap visual components to enhance the design and style of the Dash GUI.

- Pandas (import pandas as pd): Data manipulation and analysis. Read, write and transform data stored in CSV files and DataFrames to feed prediction models.

- Datetime (from datetime import datetime): Generate timestamps and work with dates for system logs and temporal analysis.

- OS (import os): Manipulate file paths and handle environment variables, such as loading CSV files.

- JSON and URLLIB (import json, urllib): Work with data in JSON format and make HTTP requests when necessary (although not used directly in the code provided).

- NumPy (import numpy as np): Perform efficient numerical calculations, especially for matrix operations in predictive models.

- PyCaret (from pycaret.classification import *): Load classification models for prediction. PyCaret facilitates the creation, evaluation and deployment of machine learning models.

- Spacy (import spacy): Natural language processing (NLP), including tokenisation, lemmatisation and stop word removal for Spanish text analysis.

- Nltk (from nltk.tokenize import word_tokenize, from nltk.stem import SnowballStemmer): Tokenisation of texts and application of stemming in text analysis.

- Scikit-learn (from sklearn.feature_extraction.text import CountVectorizer): Generate Bag of Words (BOW) representations of texts for the analysis of nutrition, alcohol, exercise and tobacco recommendations.

- SpellChecker (from spellchecker import SpellChecker): Spellcheck words in the analysed medical texts to ensure the quality of the input data.
