# Supplementary material for: Predicting diabetes mellitus metabolic goals and chronic complications transitions—analysis based on natural language processing and machine learning models
Source: PLoS One. 2025 Apr 15;20(4):e0321258. doi: 10.1371/journal.pone.0321258 (PMC11999128; doi:10.1371/journal.pone.0321258)
Supplement: S3 File — (DOCX) [file pone.0321258.s003.docx]

***Appendix 3.*** Details on the ML methods

**S3.1. Selected methods**

The selection of the Machine Learning models was based on the characteristics of the data and the nature of the problem of predicting the progression of diabetes. Following Ahsan, Luna, and Siddique (2022) and Khalilnejad et al (2024), the rational for the selected models is the following:

**1) K-Nearest Neighbors (KNN):** The KNN was selected because of its simplicity and effectiveness in scenarios with smaller or less complex data sets. Although it can be sensitive to dimensionality, it is helpful as a benchmark for comparing more complicated models.

**2) Logistic Regression (LR):** LR is a standard model widely used for binary classification problems, and its interpretation is straightforward. Since the problem of predicting diabetes progression can be decomposed into binary classifications, LR provides a good starting point for understanding the contributions of different variables.

**3) Decision Tree (DT):** DT effectively captures nonlinear interactions between variables, which is standard in medical scenarios where multiple factors influence disease progression. In addition, decision trees are easily interpretable, allowing clinicians to follow the reasoning behind the predictions.

**4) Random Forest (RF):** This algorithm is robust to overfitting and is suitable for high-dimensional clinical data with possible complex interactions between variables. RF combines multiple decision trees to improve accuracy and reduce the variability of predictions.

**5) Neural Network (NN):** NNs were selected because of their ability to model complex patterns in the data, mainly when there are nonlinear relationships that simpler models cannot adequately capture. Since the problem includes multiple variables and potentially non-trivial relationships, NNs provide a more flexible and robust architecture.

**6) Boosting:** Boosting algorithms were selected for their ability to improve accuracy by correcting errors in the base models. These algorithms are beneficial in unbalanced data sets.

**7) Training and upsampling strategies:**

A 10-fold cross-validation (StratifiedKFold) was used to evaluate each model's performance. This approach ensures that the model is trained on various data set partitions while maintaining the proportion of classes in each fold. In this case, cross-validation aimed to select hyperparameters and evaluate the model's generalization. This strategy ensures that the results reflect an average of several data set splits, providing a more robust metric. Upon completion of the ten iterations, the model's performance at each fold is averaged to obtain an overall estimate of its performance on the training data.

A complementary exercise was performed considering a test set to evaluate the results obtained from the cross-validation and to ensure the robustness of the model. The complete data is initially divided into two sets: the training set and the test set. The test set is reserved for a final evaluation and is not used at any point during the model fitting process.

The training set is divided into ten equal parts (or folds). After completing the ten iterations, the model's performance across all folds is averaged to provide an overall estimate of its performance on the training data. Following refinement through cross-validation, a final evaluation is conducted using the independent test set, which was not used during the training or validation phases.

**References**

Ahsan, M. M., Luna, S. A., & Siddique, Z. (2022). Machine-Learning-Based Disease Diagnosis: A Comprehensive Review. *Healthcare*, *10*(3), 541. https://doi.org/10.3390/healthcare10030541

Khalilnejad, A., Sun, R.-T., Kompala, T., Painter, S., James, R., & Wang, Y. (2024). Proactive Identification of Patients with Diabetes at Risk of Uncontrolled Outcomes during a Diabetes Management Program: Conceptualization and Development Study Using Machine Learning. *JMIR Formative Research*, *8*, e54373. https://doi.org/10.2196/54373
