# Supplementary material for: Predicting diabetes mellitus metabolic goals and chronic complications transitions—analysis based on natural language processing and machine learning models
Source: PLoS One. 2025 Apr 15;20(4):e0321258. doi: 10.1371/journal.pone.0321258 (PMC11999128; doi:10.1371/journal.pone.0321258)
Supplement: Attachment — Submitted filename: Authors_response_-_Plos_one_auresp_2.docx [file pone.0321258.s007.docx]

Bogotá, November 27th 2024

Dear Editor and reviewers of PLoS ONE

Once again, we thank you for your time and effort in reviewing our manuscript entitled "Predicting Diabetes Mellitus Metabolic Targets and Chronic Complications Transitions—Analysis Based on Natural Language Processing and Machine Learning Models.” Detailed responses to the reviewer’s comments are given below. In the manuscript, changes in content have been highlighted in red inside the main document.

Kind regards,

The authors

*Reviewer #4:*

***Clarity and Reproducibility:*** The manuscript would benefit from a more detailed description of the NLP preprocessing steps. For instance, specifying techniques used for text normalization (e.g., stemming, lemmatization), handling of stopwords, and dealing with medical abbreviations and jargon could improve clarity. Explaining these choices can help other researchers replicate your findings and understand the basis of your NLP pipeline’s effectiveness.

***Error Analysis:*** Discuss any challenges faced during the text preprocessing, such as misclassifications or unusual patterns in the data. Addressing how these were handled or mitigated would provide insights into the robustness of the NLP methodology.

**Justification for ML Algorithm Selection:**

***Algorithm Suitability:*** While the manuscript employs a variety of ML algorithms, there is little discussion on why these specific algorithms were chosen over others. For example, detailing the reasons for selecting neural networks or decision trees based on their suitability for the dataset characteristics (e.g., size, feature type, non-linearity) would enrich the analysis.

***Performance Comparison:*** Provide a comparative analysis of the ML algorithms used. This could include discussions on each algorithm's performance with respect to handling imbalanced data, computational efficiency, and ease of interpretation. Such comparisons could be instrumental in justifying the choice of one model over another.

**Authors’ reply (last two queries):** We agree that these elements are essential to guarantee clarity and reproducibility. Our strategy for the document was to keep it as simple as possible so it is accessible for the general public of the journal, but at the same time include an appendix that is as clear as possible. Following your recommendations, we have further enhanced the supplemental materials 2 and 3 (NLP and ML details), but also expanded the relevant section in the main text.

With respect to the selected algorithms, we discuss this in detail in the supplemental material 3. We mention that the selection was based on the characteristics of the data and the nature of the problem of predicting the progression of diabetes: **1) K-Nearest Neighbors (KNN):** The KNN was selected because of its simplicity and effectiveness in scenarios with smaller or less complex data sets. Although it can be sensitive to dimensionality, it is helpful as a benchmark for comparing more complicated models. **2) Logistic Regression (LR):** LR is a standard model widely used for binary classification problems, and its interpretation is straightforward. Since the problem of predicting diabetes progression can be decomposed into binary classifications, LR provides a good starting point for understanding the contributions of different variables. **3) Decision Tree (DT):** DT effectively captures nonlinear interactions between variables, which is standard in medical scenarios where multiple factors influence disease progression. In addition, decision trees are easily interpretable, allowing clinicians to follow the reasoning behind the predictions. **4) Random Forest (RF):** This algorithm is robust to overfitting and is suitable for high-dimensional clinical data with possible complex interactions between variables. RF combines multiple decision trees to improve accuracy and reduce the variability of predictions. **5) Neural Network (NN):** NNs were selected because of their ability to model complex patterns in the data, mainly when there are nonlinear relationships that simpler models cannot adequately capture. Since the problem includes multiple variables and potentially non-trivial relationships, NNs provide a more flexible and robust architecture. **6) Boosting:** Boosting algorithms were selected for their ability to improve accuracy by correcting errors in the base models. These algorithms are beneficial in unbalanced data sets.

We have included this discussion in the supplemental material S3.

**Model Validation Techniques:**

***Validation Strategy:*** The manuscript mentions the use of cross-validation but does not specify the type (e.g., k-fold, leave-one-out). Detailing the choice of cross-validation method, the number of folds used, and how the data was split can affect model evaluation significantly, especially in medical data where patient independence is crucial.

**Authors’ reply:** We have expanded the specific paragraph to clarify the 10-fold cross-validation (StratifiedKFold) that was used to evaluate model performance:

*This approach ensures that the model is trained on various data set partitions while maintaining the proportion of classes in each fold. In this case, cross-validation aimed to select hyperparameters and evaluate the model's generalization. Upon completion of the ten iterations, the model's performance at each fold is averaged to obtain an overall estimate of its performance on the data. This strategy ensures that the results reflect an average of several data set splits, providing a more robust metric.*

***Handling of Imbalanced Data:*** Medical datasets often have class imbalance issues, particularly in disease prediction. Discussing strategies employed to handle imbalance, such as synthetic minority oversampling technique (SMOTE) or adjusting class weights in model training, would be valuable.

**Authors’ reply:** Our dataset did present a class imbalance issue. This guided choices such as the selection of the performance metrics and the cross validation. Still, we tried to address the challenges presented by the imbalance with two main approaches - (i) increasing the proportion of the minority class with a Synthetic Minority Oversampling Technique (SMOTE), while holding the majority class constant; and (ii) increasing the minority class by means of the SMOTE and decreasing the majority class as to achieve higher balance. Although the metrics in the training set improved, the results obtained in the test set were significantly lower. This may be due to several factors, such as overfitting the model to the balanced distribution in training, which is not reflected in the actual data in the test set, or that the model may be capturing irrelevant patterns that do not generalize well to unbalanced situations, negatively affecting its performance outside the training set. This discussion is included in the supplemental material S3.

**Hyperparameter Optimization:**

***Optimization Details:*** Expanding on the hyperparameter optimization process would strengthen the methodological rigor. For instance, detailing the parameters explored, the range of values tested, and the optimization algorithm (e.g., grid search, random search, Bayesian optimization) could provide deeper insight.

***Impact on Model Performance:*** Discuss how hyperparameter tuning affected the performance of each model. This could include changes in model accuracy, sensitivity, specificity, and computational demands. Such details could help justify the final model configuration chosen for the study.

**Authors’ reply (last two queries):** We employed PyCaret to build each model’s pipeline. It initially runs a Feature Selection module that, in a nutshell, iteratively runs a LightGradientBoosting Machine model on different sets of characteristics to pick the most relevant ones for classification. In the next step, the pipeline sets up a grid of models, where each model has a parameter grid, so we perform a grid search across both models and hyperparameters to determine which is the best predictor for a specific transition and outcome. For instance, after picking the most relevant predictors, we set up a parameter grid for logistic regression and performed cross-validation across all the parameter grid points. This process is done for all the available models - roughly 19 options -  and the one that maximizes the F1 is finally chosen as the best predictor. Naturally, having so many nested grid searches (across variables, models and hyperparameters) leads to a higher computational complexity.

**Performance Metrics Explanation:**

***Metric Selection Rationale:*** While accuracy, F1 score, and AUC are standard, discussing why these were chosen and their relevance to the study’s clinical implications would be insightful. For instance, in a medical context, the cost of false negatives might be more critical than false positives.

**Authors’ reply:** In the relevant subsection of the methods section we introduced a paragraph with the following explanation:

*Considering the performance metrics, a common scenario when considering imbalanced datasets is that the F1 score exhibits proficiency, while the AUC appears suboptimal. The AUC can yield a lower value due to challenges in effectively distinguishing the minority class amidst the dominance of the majority class. Supplemental material 3 includes more details about the process and alternative strategies that we considered.*

In addition, a further exercise was conducted to balance the training sample by increasing the proportion of the minority class or balancing both classes. Although the metrics in the training set improved, the results obtained in the test set were significantly lower. This may be due to several factors, such as overfitting the model to the balanced distribution in training, which is not reflected in the actual data in the test set, or that the model may be capturing irrelevant patterns that do not generalize well to unbalanced situations, negatively affecting its performance outside the training set. See supplemental material S3 for further details.

Table 6 presents the best algorithm for each of the 12 models. The entire set of metrics over all tested algorithms is presented in supplemental material S4. We privilege F1 due to the imbalanced nature of our data. We introduced the following paragraph after presenting the metrics in the results section:

*As discussed in the methods section, in imbalanced datasets, the F1 score gives favorable results when the predictive model excels in identifying the less prevalent but critical class (outside goals or with complications)*

***Thresholds and Trade-offs:*** Elaborate on any thresholds used for classification decisions (e.g., cut-off probabilities for classifying a patient’s disease stage). Discussing the trade-offs between sensitivity and specificity in choosing these thresholds, especially how they might impact clinical decisions, would be extremely beneficial.

**Authors’ reply:** Thresholds were employed in two stages of the study - (i) when classifying the patient's HbA1c levels and comorbidities, hence the initial states and the outcomes, and (ii) when predicting the patients’ transitions. For the former classification, we employed both the ICD-10 codes used in the electronic health record (EHR) system and an additional verification process. Specifically, we utilized ICD-10 codes to identify patients diagnosed with diabetes mellitus type 1 or type 2. Furthermore, to ensure accuracy, the status of these diagnoses was cross-checked by the auditing department of the Health Maintenance Organization (HMO). This dual approach helped us confirm the validity of the diagnoses included in our study. For the latter classification, which was done by the machine learning models, we were agnostic when defining a rule to optimize the cutoff probability. Nevertheless, an ideal scenario could be to set the cutoff to maximize sensitivity - i.e., patients are correctly classified either as developing comorbidities or deviating from their ideal HbA1c levels, such that practitioners can try to prevent these outcomes.
