# Supplementary material for: Predicting diabetes mellitus metabolic goals and chronic complications transitions—analysis based on natural language processing and machine learning models
Source: PLoS One. 2025 Apr 15;20(4):e0321258. doi: 10.1371/journal.pone.0321258 (PMC11999128; doi:10.1371/journal.pone.0321258)
Supplement: Attachment — Submitted filename: Authors response - Plos one.docx [file pone.0321258.s006.docx]

Bogotá, September 11th 2024

Dear Editor and reviewers of PLoS ONE

We thank you for your time and effort in reviewing our manuscript entitled "Predicting Diabetes Mellitus Metabolic Targets and Chronic Complications Transitions—Analysis Based on Natural Language Processing and Machine Learning Models.” Detailed responses to reviewer’s comments are given below. In the manuscript, changes in content have been highlighted in red inside the main document.

Kind regards,

The authors

*Journal Requirements: When submitting your revision, we need you to address these additional requirements.*

*1. Please ensure that your manuscript meets PLOS ONE's style requirements, including those for file naming. The PLOS ONE style templates can be found at* 
[*https://journals.plos.org/plosone/s/file?id=wjVg/PLOSOne_formatting_sample_main_body.pdf*](https://journals.plos.org/plosone/s/file?id=wjVg/PLOSOne_formatting_sample_main_body.pdf) *and* 
[*https://journals.plos.org/plosone/s/file?id=ba62/PLOSOne_formatting_sample_title_authors_affiliations.pdf*](https://journals.plos.org/plosone/s/file?id=ba62/PLOSOne_formatting_sample_title_authors_affiliations.pdf)

**Authors reply:** We have carefully revised the style requirements. Hopefully the manuscript is in line with those requirements in this revision.

*2. Please note that PLOS ONE has specific guidelines on code sharing for submissions in which author-generated code underpins the findings in the manuscript. In these cases, we expect all author-generated code to be made available without restrictions upon publication of the work. Please review our guidelines at* [*https://journals.plos.org/plosone/s/materials-and-software-sharing#loc-sharing-code*](https://journals.plos.org/plosone/s/materials-and-software-sharing#loc-sharing-code) *and ensure that your code is shared in a way that follows best practice and facilitates reproducibility and reuse.*

**Author's reply:** The code required for reproducibility and reuse will be uploaded to a GitHub repository upon publication.

*3. We note that the grant information you provided in the ‘Funding Information’ and ‘Financial Disclosure’ sections do not match. When you resubmit, please ensure that you provide the correct grant numbers for the awards you received for your study in the ‘Funding Information’ section.*

**Authors reply:** This is our funding information “This study was supported by the Ministry of Science, Technology and Innovation (Minciencias) of Colombia. Grant Number: 138-2021. Andrés García and Paul Rodríguez acknowledge support by Fulbright-Colciencias and Colombia Cientifica – Alianza EFI 60185 contract FP44842- 220-2018, funded by The World Bank through the Scientific Ecosystems, managed by the Colombian Ministry of Science, Technology and Innovation (MINCIENCIAS).”

*4. Thank you for stating the following financial disclosure: "This study was supported by the Ministry of Science, Technology and Innovation (Minciencias) of Colombia. Grant Number: 138-2021, Universidad del Rosario, Fundación Universitaria Sanitas, and Universidad EIA in Colombia. Andrés García and Paul Rodríguez acknowledge support by the Ministry of Science, Technology and Innovation (Minciencias) of Colombia. Grant Number: 138-2021"*

**Authors reply:** We updated the financial disclosure to reflect that this study was supported by the Ministry of Science, Technology and Innovation (Minciencias) of Colombia. Grant Number: 138-2021. Andrés García and Paul Rodríguez acknowledge support by Fulbright-Colciencias and Colombia Cientifica – Alianza EFI 60185 contract FP44842- 220-2018, funded by The World Bank through the Scientific Ecosystems, managed by the Colombian Ministry of Science, Technology and Innovation (MINCIENCIAS). The funders had no role in study design, data collection and analysis, decision to publish, or manuscript preparation.

*5. Please state what role the funders took in the study.  If the funders had no role, please state: "The funders had no role in study design, data collection and analysis, decision to publish, or preparation of the manuscript." If this statement is not correct you must amend it as needed. 
Please include this amended Role of Funder statement in your cover letter; we will change the online submission form on your behalf.*

**Authors reply:** We have updated the statement in the document and the cover letter.

*6. Thank you for stating the following in your Competing Interests section:  "None"
 
Please complete your Competing Interests on the online submission form to state any Competing Interests. If you have no competing interests, please state ""The authors have declared that no competing interests exist."", as detailed online in our guide for authors at* [*http://journals.plos.org/plosone/s/submit-now*](http://journals.plos.org/plosone/s/submit-now)  *This information should be included in your cover letter; we will change the online submission form on your behalf.*

**Authors reply:**  We have updated the statement in the document and the cover letter.

*6. When completing the data availability statement of the submission form, you indicated that you will make your data available on acceptance. We strongly recommend all authors decide on a data sharing plan before acceptance, as the process can be lengthy and hold up publication timelines. Please note that, though access restrictions are acceptable now, your entire data will need to be made freely accessible if your manuscript is accepted for publication. This policy applies to all data except where public deposition would breach compliance with the protocol approved by your research ethics board. If you are unable to adhere to our open data policy, please kindly revise your statement to explain your reasoning and we will seek the editor's input on an exemption. Please be assured that, once you have provided your new statement, the assessment of your exemption will not hold up the peer review process.*

**Authors reply:**  We have included the following code and data sharing statement:

All the code required to reuse the algorithms and replicate the results with the original data will be available on GitHub upon publication.

The original data that support the findings of this study are available from HMO EPS Sanitas. Restrictions apply to the availability of these data due to the involvement of sensible personal data (electronic health records), which were used under license for this study and under the authorization of the Unisanitas Ethics and Research Board. Any researcher that would like to get access to the dataset will be required to apply for access by discussing it with Unisanitas and HMO EPS Sanitas research offices: investigaciones@unisanitas.edu.co

*7. Please include captions for your Supporting Information files at the end of your manuscript, and update any in-text citations to match accordingly. Please see our Supporting Information guidelines for more information:* [*http://journals.plos.org/plosone/s/supporting-information*](http://journals.plos.org/plosone/s/supporting-information)*.*

**Authors reply:** We have added the corresponding captions

*Reviewer #1: Summary:*

*In this study, the authors use natural language processing (NLP) and machine learning (ML) to predict diabetes progression from structured and unstructured electronic health records (EHRs).*

*Comment 1: - The authors state that patients with a confirmed diagnosis of diabetes were included. Is confirmed diagnosis referred to ICD codes or specific diagnosis definitions used by the EHR system? Were only patients with type 2 diabetes included or also with type 1 diabetes?*

**Authors reply:** For the study, the term "confirmed diagnosis" refers to both the ICD-10 codes used in the electronic health record (EHR) system and an additional verification process. Specifically, we utilized ICD-10 codes to identify patients with a diagnosis of diabetes mellitus type 1 or type 2. Furthermore, to ensure accuracy, the status of these diagnoses was cross-checked by the auditing department of the Health Maintenance Organization (HMO). This dual approach helped us confirm the validity of the diagnoses included in our study.

We included this discussion at the start of the research design and methods section.

*Comment 2*

*- Why did the authors choose not to include neuropathy, peripheral vascular disease, and cardiovascular disease as complications?*

**Authors reply:** The authors chose not to include neuropathy and peripheral vascular disease due to the limited number of incident cases observed during the follow-up period (see table below). In practice, the inclusion or not of such other complications have little relevance on the empirical results as the vast majority of complications correspond to CKD. Not including it was relevant as the incidence of the other cardiovascular conditions tends to be higher in other populations. Hence, readers might consider that these conditions are a common reason behind the state transition, but this is not the case in our data. Thus, to avoid such misleading conclusion, we preferred to explicitly exclude them from the status definition.

*Comment 3*

*- How were the complications defined? For chronic kidney disease, were also estimated glomerular filtration rates considered?*

**Authors reply:** In the supplemental material, appendix 1 presents the ICD-10 codes that define the complications. In the case of CKD, we also use GFR to classify the CKD status.

*Comment 4*

*- The authors state that they used cross-validation to find the best performing model. However, they did not provide any information on whether cross-validation was just used for hyperparameter tuning or whether a nested cross-validation scheme was used to also get unbiased estimates of the models’ performance. Further, no information on the number of folds was provided.*

**Authors reply:** A 10-fold cross-validation (StratifiedKFold) was used to evaluate model performance. This approach ensures that the model is trained on various data set partitions while maintaining the proportion of classes in each fold. In this case, cross-validation aimed to select hyperparameters and evaluate the model's generalization. This strategy ensures that the results reflect an average of several data set splits, providing a more robust metric. Upon completion of the ten iterations, the model's performance at each fold is averaged to obtain an overall estimate of its performance on the data.

We added this information to the Supplemental Material S3.

*Comment 5*

*- The results suggest that the ML models did not learn anything during training. For a binary classifier to achieve an F1-score of 0.95 and an AUC of 0.5, the dataset has to be very imbalanced (as is the case in the paper) and the predicted probabilities must be all very close to zero or one (depending whether the majority class labels are zero or one). The authors should carefully check their ML pipeline again.*

Authors reply: Considering the performance metrics, the F1 score exhibits proficiency, while the AUC appears suboptimal. This phenomenon is notably observed in imbalanced datasets, where the instances of one class significantly outnumber those of the other. In our case, the F1 score gives favorable results when the predictive model identifies the less prevalent but critical class (outside goals or with complications). However, the AUC can yield a lower value due to challenges in effectively distinguishing the minority class amidst the dominance of the majority class (within goals or without complications). This means that the discrepancies between these indicators' values are due to how they are mathematically calculated.

In addition, a further exercise was conducted to balance the training sample by increasing the proportion of the minority class or balancing both classes. Although the metrics in the training set improved, the results obtained in the test set were significantly lower. This may be due to several factors, such as overfitting the model to the balanced distribution in training, which is not reflected in the actual data in the test set, or that the model may be capturing irrelevant patterns that do not generalize well to unbalanced situations, negatively affecting its performance outside the training set. See supplemental material S3 for further details.

*Comment 6*

*- Mean HbA1c levels are not reported in Table 1.*

**Authors reply:** Many thanks for pointing this out. This information was added to the table.

*Comment 7*

*- I would expect the authors to provide the full list of covariates that were used for the ML models.*

**Authors reply:** We have improved the description of the available information in the dataset but also made it clear which variables are used for the estimation of the model and how they were constructed. This is presented in the new Table 1.

*Comment 8*

*- I would suggest removing Figure 1. It is clear from the text what is meant and the Figure does not look professional enough for a scientific publication.*

**Authors reply:** We have modified the figure, so it is a bit closer to the classic designs of similar diagrams in the literature. Although the model is quite simple, we still believe it helps readers to follow the possible transitions.

*Comment 9*

*- I am not an expert in NLP, thus I cannot assess whether the bag-of-words method is a reasonable choice for the task at hand.*

**Authors reply:** We have rewritten the section in order to clarify the goal of the task and why the bags of word method is ideal. We brought from the supplementary material a simple example that helps to illustrate the idea.

*Reviewer #2: This is a generally solid study in terms of both methodology and manuscript quality. The concept is interesting and somewhat unique within the broader landscape of published research. However, there are some areas where the manuscript could be improved, particularly by addressing certain missing details in the methodology:*

*Methods:*

*Population Description: The description of the population lacks specifics regarding inclusion and exclusion criteria. For example, the phrase "patients with a confirmed diagnosis of DM" is vague. It would be helpful to specify the diagnostic criteria used for DM and clarify whether certain patient groups (e.g., those with a recent diagnosis, pregnancy, etc.) were excluded from the study.*

**Authors reply:** For the study, the term "confirmed diagnosis" refers to both the ICD-10 codes used in the electronic health record (EHR) system and an additional verification process. Specifically, we utilized ICD-10 codes to identify patients with a diagnosis of diabetes mellitus type 1 or type 2. Furthermore, to ensure accuracy, the status of these diagnoses was cross-checked by the auditing department of the Health Maintenance Organization (HMO). This dual approach helped us confirm the validity of the diagnoses included in our study.

We included this discussion at the start of the research design and methods section.

*NLP Methodology: The Natural Language Processing (NLP) techniques described require more detailed explanation regarding the algorithms and parameters used. For instance, while you mention the creation of a custom medical dictionary, it is unclear how this was integrated into the NLP pipeline (e.g., which specific NLP models or tools, such as SpaCy, NLTK, etc., were utilized). Additionally, provide clarification on how the accuracy of the labels generated by the NLP methods was validated.*

**Authors reply:** We have rewritten the section to better illustrate the procedure. In addition, supplementary material 2 (S2) includes the detailed explanation of the procedure and a list of all the libraries integrated into the NLP pipeline.

It is important to mention that given our usage of NLP, we do not have metrics for the NLP as we use it as a data processing step. In this sense, every time that our clinical experts reviewed the classification results, we corrected the dictionary to improve the results. In this sense, any ex-post construction of a metric would be uninformative as it is the result of iterating over the same dataset. Ideally, the metrics should be obtained by the revision of the classifications by an independent clinical expert team in another dataset. We have added this as a suggestion for future research in the limitations’ paragraph.

*Model Performance Metrics: The results section mentions F1 scores and accuracy for different models but lacks discussion on how these metrics compare across models and the reasons certain models outperformed others. Including a table summarizing the performance metrics for each model, along with a brief discussion of the strengths and weaknesses of each approach, would enhance the clarity of the findings.*

**Authors reply:** Table 6 presents the best algorithm for each of the 12 models. The entire set of metrics over all tested algorithms is presented in supplemental material S4. We privilege F1 due to the imbalance nature of our data. We introduced the following paragraph after presenting the metrics in the results section:

“Considering the performance metrics, the F1 score exhibits proficiency, while the AUC appears suboptimal. This phenomenon is notably observed in imbalanced datasets, where the instances of one class significantly outnumber those of the other. In our case, the F1 score gives favorable results when the predictive model excels in identifying the less prevalent but critical class (outside goals or with complications). However, the AUC can yield a lower value due to challenges in effectively distinguishing the minority class amidst the dominance of the majority class (within goals or without complications). “

This means that the discrepancies between the values of these indicators are due to the way they are mathematically calculated.

*ML Model Selection: The manuscript references several machine learning algorithms (KNN, LR, DT, RF, NN, Boosting) but does not provide a rationale for selecting these specific models. Offering a brief justification for each model's selection based on the nature of the data and the research problem would strengthen the rigor of the methodology section. Additionally, more details on the hyperparameter optimization process and how it was tailored to each model would be beneficial.*

**Authors reply:** The selection of the Machine Learning models was not arbitrary. Still, it was based on the characteristics of the data and the nature of the problem of predicting the progression of diabetes: **1) K-Nearest Neighbors (KNN):** The KNN was selected because of its simplicity and effectiveness in scenarios with smaller or less complex data sets. Although it can be sensitive to dimensionality, it is helpful as a benchmark for comparing more complicated models. **2) Logistic Regression (LR):** LR is a standard model widely used for binary classification problems, and its interpretation is straightforward. Since the problem of predicting diabetes progression can be decomposed into binary classifications, LR provides a good starting point for understanding the contributions of different variables. **3) Decision Tree (DT):** DT effectively captures nonlinear interactions between variables, which is standard in medical scenarios where multiple factors influence disease progression. In addition, decision trees are easily interpretable, allowing clinicians to follow the reasoning behind the predictions. **4) Random Forest (RF):** This algorithm is robust to overfitting and is suitable for high-dimensional clinical data with possible complex interactions between variables. RF combines multiple decision trees to improve accuracy and reduce the variability of predictions. **5) Neural Network (NN):** NNs were selected because of their ability to model complex patterns in the data, mainly when there are nonlinear relationships that simpler models cannot adequately capture. Since the problem includes multiple variables and potentially non-trivial relationships, NNs provide a more flexible and robust architecture. **6) Boosting:** Boosting algorithms were selected for their ability to improve accuracy by correcting errors in the base models. These algorithms are beneficial in unbalanced data sets.

We have included this discussion in the supplemental material S3.

*Handling of Missing Data: There is no discussion of how missing data were addressed, particularly in the EHR records. It would be helpful to specify the approach taken (e.g., imputation methods, exclusion of incomplete cases) and discuss the potential impact of missing data on the study’s results.*

**Authors reply:**

We did not impute missing data as it was uncommon in the data, but there is a central element that should be highlighted due to its relevance for understanding a central limitation of the study (selection bias):

As presented in the text, the main filter for selecting our sample was the availability of at least one measurement of HbA1C for three consecutive years (2018, 2019, and 2020). This filter alone reduced the sample from 75,714 patients o 25,320. After this, we filter for having information on the predictors (other biomarkers and demographic information). Such selection reduced the sample to 23,802 patients. Hence, the central selection process is determined by the availability of HbA1C, which is used to define the IN-goals and OUT-goals states. As this variable is used to define a central outcome of the model, imputing it might substantially bias the analysis. Missings in the other variables are quite uncommon as these are administrative records, and other measurements are usually captured at the same time as the HbA1C (for instance, cholesterol levels).

We amended the start of the results section to make this point clear:

“The original dataset started with 75,714 patients. Of those, only 25,320 had HbA1C measurements in the three study years, even though CPG suggest at least two measurements per year. Of those, a total 23,802 patients were included in the analysis as the remainder had missing information in some the predictor variables.”

Discussion:

*The conclusion discusses the potential impact of the study but could be more specific. For instance, you mention the possibility of "improved health outcomes" without specifying which outcomes might be most affected. Consider providing concrete examples of how the findings could influence clinical practice, such as specific interventions that could be implemented*

**Authors reply:** we have revised the conclusion to include concrete examples of how the findings could influence clinical practice**.** We expanded the specific paragraph as follows:

“This study could impact patients and the healthcare system from a clinical perspective. The model developed might aid in identifying individuals at risk of future complications early, enabling timely preventive measures. We highlight that the incidence of complications, despite achieving glycemic control, suggests the need for more comprehensive management strategies in patients with diabetes mellitus. For example, healthcare providers could implement more frequent monitoring of renal function (as indicated by the glomerular filtration rate) and cardiovascular health, especially in older patients or those with a history of poor compliance to treatment, even when they are within HbA1c targets. Additionally, our findings support the importance of reinforcing adherence to treatment guidelines for HbA1c and dyslipidemia, as well as personalized patient education focusing on lifestyle interventions, such as diet and exercise. These interventions could lead to better management of chronic complications like cardiovascular disease and kidney failure, ultimately improving long-term health outcomes in this population.”

*Limitations: The limitations section is somewhat underdeveloped. While you mention a short follow-up period and the lack of data on hospitalizations or mortality, other potential limitations, such as selection bias, the accuracy of NLP-generated labels, and the generalizability of the findings to other populations, are not addressed. Expanding on these aspects will provide a more comprehensive assessment of the study's limitations.*

**Authors reply:** We have now acknowledged this limitation and emphasized the need for caution when interpreting the findings in broader clinical settings. Also, we added a note about the absence of external validation and highlighted the need for future studies to replicate our findings in diverse populations to assess their broader applicability. The limitations’ paragraph was amended as follows:

“This analysis has several limitations. First, DM is a chronic disease usually related to complications incidence for five years, and our follow-up considers only 1 and 2 years, which is too short to identify all new cases in the cohort. Our model focuses on measuring the progression of DM towards critical stages like the development of complications. However, we had no data on more decisive outcomes such as hospitalization and mortality. Second, we recognize that the retrospective nature of our study and the inclusion criteria based on the availability of HbA1c measurements could introduce selection bias. Patients with more frequent follow-ups or better adherence to treatment might be overrepresented, potentially influencing the results. In addition, if the patients with diabetes who moved were more (or less) likely to develop complications or to be out of the sample, our selection would induce a bias. The usual transfer rate of general patients between HMOs in the country was around 6%^20^, but we do not have such data for the HMO studied. Third, as our study was conducted within a single HMO in Colombia, the findings may not be directly generalizable to other populations or healthcare systems. Elements such as the NLP-generated labels are typically specific to the Colombian context, and to some extent to the specific HMO. Still, the general procedure can be replicated elsewhere. For future work, we suggest validating the NLP exercise by obtaining metrics through the revision of the classifications by an independent clinical expert team in another dataset. In general, we recommend validating the presented prediction model in other populations prior to its clinical use. “

*General:*

*Consistency in Terminology: Ensure consistency in terminology throughout the manuscript. For example, the terms "out of targets" and "outside goals" are used interchangeably in different sections. Standardizing the language will help avoid confusion and improve readability.*

**Authors reply:** We are now using “goals” as the standard; we keep still one mention of targets as it is a relevant keyword. Also, we had a similar issue with “comorbidities” and “complications”; now we are using the term “complications” only.

*Conclusion: Incorporating more detailed explanations of the methodology and discussing the practical implications of the study in day-to-day clinical practice could significantly enhance the value of this already valuable study.*

**Authors reply:** We have also expanded the discussion of how the study's findings can be applied in day-to-day clinical practice. We added the following paragraph:

Our results indicate that closer monitoring of patients within HbA1c targets but at risk of complications (such as those with declining renal function) could lead to earlier interventions. Moreover, reinforcing adherence to both pharmacological treatments and lifestyle modifications may prevent further progression of the disease. These practical recommendations, based on our model’s predictions, could assist healthcare providers in tailoring patient care, thus improving long-term health outcomes.

*Reviewer #3:*

*Journal Style and Format: At first glance, the current context of your paper does not align with the typical structure of a journal article. It reads more like a summary report with limited data. Please ensure that your manuscript adheres to the journal’s style guidelines.*

**Authors reply:** Many thanks for the advice. We have carefully reviewed the guidelines of this journal in order to comply with them.

*Literature Review: The literature review is insufficient. Typically, a robust review should include around 20 related articles focusing on the application of ML and NLP in disease diagnosis. Your review lacks depth and does not cover trends in disease complications. Please review at least 10 journal articles in your field that utilize machine learning and provide a comparative table of their results.*

**Authors reply:** Ideally, we would have such articles available; however, when the study started, we found few articles available as most of the diabetes-ML literature with HER data is about predicting the onset of the condition, not on its progression.

We have redone the literature review using PubMed. We searched for “diabetes progression machine learning” and retrieved 464 articles from 2009 to 2024. After revising titles, 15 were selected; one of them the article that we used as a comparison. After revising the abstract, 7 of them are related to our study; aside from on 2019 paper, all of them were published since 2021 (see table below). We included these documents into our paper discussion. We did not include the suggested the table as the outcomes are not exactly the same across papers, which limits the standardization of the comparisons.

| Title | Authors | Journal and issue |
| --- | --- | --- |
| Artificial intelligence predicts the progression of diabetic kidney disease using big data machine learning | Makino M, Yoshimoto R, Ono M, Itoko T, Katsuki T, Koseki A, Kudo M, Haida K, Kuroda J, Yanagiya R, Saitoh E, Hoshinaga K, Yuzawa Y, Suzuki A. | Sci Rep. 2019 Aug 14 |
| Development and internal validation of machine learning algorithms for end-stage renal disease risk prediction model of people with type 2 diabetes mellitus and diabetic kidney disease | Zou Y, Zhao L, Zhang J, Wang Y, Wu Y, Ren H, Wang T, Zhang R, Wang J, Zhao Y, Qin C, Xu H, Li L, Chai Z, Cooper ME, Tong N, Liu F. | Ren Fail. 2022 Dec |
| Derivation and validation of a machine learning risk score using biomarker and electronic patient data to predict progression of diabetic kidney disease | Chan L, Nadkarni GN, Fleming F, McCullough JR, Connolly P, Mosoyan G, El Salem F, Kattan MW, Vassalotti JA, Murphy B, Donovan MJ, Coca SG, Damrauer SM. | Diabetologia. 2021 Jul |
| Predicting Progression of Type 2 Diabetes Using Primary Care Data with the Help of Machine Learning | Ozturk B, Lawton T, Smith S, Habli I. | Stud Health Technol Inform. 2023 May 18 |
| Predicting the 2-Year Risk of Progression from Prediabetes to Diabetes Using Machine Learning among Chinese Elderly Adults   - Already included in the previous version | Liu Q, Zhou Q, He Y, Zou J, Guo Y, Yan Y. | J Pers Med. 2022 Jun 27 |
| Machine Learning Prediction Models for Chronic Kidney Disease Using National Health Insurance Claim Data in Taiwan | Krishnamurthy S, Ks K, Dovgan E, Luštrek M, Gradišek Piletič B, Srinivasan K, Li YJ, Gradišek A, Syed-Abdul S. | Healthcare (Basel). 2021 May 7 |
| Prediction models for earlier stages of chronic kidney disease | Alexiuk M, Tangri N. | Curr Opin Nephrol Hypertens. 2024 May 1 |

*NLP Methods: You mention the implementation of NLP but do not specify which methods were used. There are three main NLP methods available. Please clarify which ones were implemented in your study.*

**Authors reply:** We have rewritten the section in order to make it easy to understand the procedure. For NLP we had three steps: pre-processing, characterization, and classification. Each step includes several procedures which are detailed in Supplemental Material 2.

*NLP Metrics: You mentioned using two approaches, simple search and BoW, and ultimately selecting BoW. However, you did not report any clear metrics. If these metrics are in the appendix, please note that the appendix is not the appropriate place for them. Include clear metrics in the main text.*

**Authors reply:** We have rewritten the section and introduced Table 1 to make clear where NLP was used. We did not run a race between simple search and BoW but rather considered in which scenarios it made more sense to use one or the other. The simple search was useful for medications and referrals, where terms are highly standardized. For more complex scenarios, like determining if a patient was adherent to the pharmacological intervention, the BoW method was used and improved with clinical experts' revisions.

*Machine Learning Methods: In the materials and methods section, you refer to both regression and classification methods, but your study focuses on classification. This makes the mention of regression methods unnecessary. It appears you implemented only neural networks but reported other methods as well. Please clarify which methods were implemented in sufficient detail.*

**Authors reply:** We are only using classification methods. The logistic regression is used as a classification method, which might be the reason of the confusion. The new supplemental material 3 describes in more details the implemented methods. In addition, supplemental material 4 presents the metrics for the 180 models that were tested for the analysis. As you can see there, we tried all the methods described but the neural networks appeared as the top performer in many scenarios; this is the reason why these methods are highlighted in Table 6.

*Results and Discussion: For NLP, it is necessary to provide a table of metrics to compare all implemented groups, including data size (optional) and accuracy. Is this information in Table 2? Please clarify.*

**Authors reply:**  Given our usage of NLP, we do not have metrics for the NLP as we use it as a data processing step. In this sense, every time that our clinical experts reviewed the classification results, we corrected the dictionary to improve the results. In this sense, any ex-post construction of a metric would be uninformative as it is the result of iterating over the same dataset. Ideally, the metrics should be obtained by the revision of the classifications by an independent clinical expert team in another dataset. We have added this as a suggestion for future research in the limitations’ paragraph.

*Classification Results: Clarify which classification method performed best and provide a consistent conclusion about the potential of your study, reducing unnecessary parts.*

**Authors reply:** Table 6 presents the models that performed best. The table presents 12 models as we estimate independent models for explaining transitions into OUT-goals and for YES-complications; we also allowed for independent models according to the initial conditions. Such structure was adopted in order to improve the fit of the overall model which had a bad performance when considering a unique model covering all potential transitions and initial conditions at once.

*Figures: Please split Figure 2 into three separate figures, as it contains a lot of information. Combining them into one figure may reduce their quality.*

**Authors reply:** Agree. We have split the figure into Figures 2 to 4.
