# Supplementary material for: Predicting diabetes mellitus metabolic goals and chronic complications transitions—analysis based on natural language processing and machine learning models
Source: PLoS One. 2025 Apr 15;20(4):e0321258. doi: 10.1371/journal.pone.0321258 (PMC11999128; doi:10.1371/journal.pone.0321258)
Supplement: Attachment — Submitted filename: Authors_response_-_Plos_one_auresp_3.docx [file pone.0321258.s008.docx]

Bogotá, February 1st 2025

Dear Editor and reviewers of PLoS ONE

We sincerely appreciate the time and effort you have dedicated to reviewing our manuscript, "Predicting Diabetes Mellitus Metabolic Targets and Chronic Complications Transitions—Analysis Based on Natural Language Processing and Machine Learning Models." Below, we provide detailed responses to the reviewer’s comments. All content modifications have been highlighted in red within the main document.

Kind regards,

The authors

Reviewer #4:

**1. Employ Additional Visual Representations:**

**Include one or two essential graphs illustrating machine learning performance, such as: A confusion matrix for the optimal model to illustrate prediction accuracy for each class. A bar chart illustrating the F1-scores of various models to facilitate comparisons for readers.**

Response: we have constructed the suggested bar chart (Figure 7) to complement Table 6, and the confusion matrix (Table 7). The confusion matrix was useful to better highlight the specific limitations that the model face.

**2. Emphasize Feature Significance: Incorporate a basic feature importance plot for a primary machine learning model (e.g., Random Forest or Logistic Regression) to illustrate which factors exerted the most significant influence on predictions.**

Response: Feature importance plots for the models in rows four and six of Table 6 are generated, these are the new Figures 3 and 4. We describe them and their implications in the main results.

**3. Concisely Elucidate Model Selections: Incorporate a brief paragraph into the main text (citing Appendix 3) that elucidates the rationale for the selection of specific machine learning models, highlighting the interpretability of Logistic Regression and the robustness of Random Forest.**

**Response:** We have incorporated the following paragraph into the manuscript to elucidate the rationale for our model selection:

*"The selection of ML models was guided by the characteristics of the dataset and the complexity of predicting diabetes progression. Specifically, LR was included due to its interpretability, making it a valuable tool for understanding the impact of individual predictors in binary classification tasks. Additionally, RF was chosen for its robustness to overfitting and ability to handle high-dimensional clinical data with complex interactions, which are prevalent in disease progression modeling. These models complement other approaches, such as KNN for benchmarking, DT for interpretability, NN for capturing nonlinear relationships and Boosting methods for handling imbalanced datasets. Supplemental material S3 provides a detailed justification for selecting each model."*

**4. Elucidate Data Preprocessing:**

Response: Concise preprocessing measures, including as oversampling and feature selection, were implemented to ensure the models adeptly managed unbalanced input. These are briefly mentioned in the “deriving structured data from free text fields” section and carefully described in supplemental material S2.

**5. Concentrate on Principal Outcomes: Identify a significant discovery from the machine learning models, such as the primary predictor for complications or metabolic objectives, and its correlation with clinical observations.**

Response: We have carefully reviewed the results and identified two central findings. First, at two years, more than 60% of patients who start in the best-case scenario—on-target HbA1c and no complications—develop chronic complications, highlighting the progressive nature of diabetes despite initial metabolic control. Second, following dyslipidemia treatment guidelines reduces the likelihood of being outside HbA1c goals and lowers the risk of complications, while non-adherence to pharmacological treatments significantly increases the probability of developing complications. These findings suggest that maintaining HbA1c within target ranges alone is not enough to prevent disease progression. Instead, they emphasize the need for enhanced monitoring, stricter adherence to lipid and kidney management guidelines, and patient education on lifestyle changes to mitigate long-term complications.

We have modified the conclusions of the document to highlight these ideas. We greatly appreciate the comment that motivated us to improve the central message.

**6. Examine Constraints Gently:**

**Response**: In our conclusions section we summarize the central limitations and restrictions from our analysis:

1) The horizon of the models, 1 and 2 years, might be too short to detect impacts on complications incidence. As a result, for the model is hard to accurately predict observed cases.
2) Attrition and missing data might introduce bias and limit external validity, which itself faces a key restriction and is that all data comes from one insurance company (although large and present in several region markets with diverse user profiles).
3) The imbalanced data that typically results on overfitting of the bigger categories. This restriction was partially mitigated using approaches such as NLP and oversampling. Still, the AUC appears suboptimal and for some models the true negatives rate is not ideal.
4) Feature importance and computed marginal effects should be taken with the perspective of associations. There is no causality relationships in this analysis. This limits the capability to provide detailed treatment recommendations.

From the outset, we aimed to establish these constraints upfront to convey a clear message: this study leverages the best available tools and highly granular patient-level data from a major insurance company over several years. Yet, despite these advantages, our ability to predict the medium-term progression of diabetes remains far from ideal.

Two key takeaways emerge: first, the current findings should be translated into practical applications, such as a calculator or decision-support tool, to assess their impact on treatment outcomes. Second, enhancing the quality and scope of available data is crucial for improving future forecasting efforts.
